# High-pressure synthesis of dysprosium carbides

**DOI:** 10.3389/fchem.2023.1210081

**Published:** 2023-06-13

**Authors:** Fariia Iasmin Akbar, Alena Aslandukova, Andrey Aslandukov, Yuqing Yin, Florian Trybel, Saiana Khandarkhaeva, Timofey Fedotenko, Dominique Laniel, Maxim Bykov, Elena Bykova, Natalia Dubrovinskaia, Leonid Dubrovinsky

**Affiliations:** ^1^ Material Physics and Technology at Extreme Conditions, Laboratory of Crystallography, University of Bayreuth, Bayreuth, Germany; ^2^ Bayerisches Geoinstitut, University of Bayreuth, Bayreuth, Germany; ^3^ State Key Laboratory of Crystal Materials, Shandong University, Jinan, China; ^4^ Department of Physics, Chemistry and Biology (IFM), Linköping University, Linköping, Sweden; ^5^ Deutsches Elektronen-Synchrotron DESY, Hamburg, Germany; ^6^ Centre for Science at Extreme Conditions and School of Physics and Astronomy, University of Edinburgh, Edinburgh, United Kingdom; ^7^ Institute of Inorganic Chemistry, University of Cologne, Cologne, Germany

**Keywords:** high-pressure, diamond anvil cell, rare-earth carbides, carbides, rare-earth elements, lanthanides carbides, dysprosium carbide

## Abstract

Chemical reactions between dysprosium and carbon were studied in laser-heated diamond anvil cells at pressures of 19, 55, and 58 GPa and temperatures of ∼2500 K. *In situ* single-crystal synchrotron X-ray diffraction analysis of the reaction products revealed the formation of novel dysprosium carbides, Dy_4_C_3_ and Dy_3_C_2_, and dysprosium sesquicarbide Dy_2_C_3_ previously known only at ambient conditions. The structure of Dy_4_C_3_ was found to be closely related to that of dysprosium sesquicarbide Dy_2_C_3_ with the Pu_2_C_3_-type structure. *Ab initio* calculations reproduce well crystal structures of all synthesized phases and predict their compressional behavior in agreement with our experimental data. Our work gives evidence that high-pressure synthesis conditions enrich the chemistry of rare earth metal carbides.

## 1 Introduction

Carbides are important compounds in science and technology due to their useful chemical, mechanical, electrical, magnetic, and optical properties (Sakai et al., 1981a; Davaasuren et al., 2010; Lengauer, 2012). The structure, bonding, and phase transitions of lanthanide carbides are of interest due to their potential applications in mechanical and electrical devices. Structural variations at high-pressure conditions can lead to a sharp change in the properties and unusual crystal chemistry of carbides.

Metal carbides containing [C_2_]^2−^ ion are known for many elements at ambient conditions (for instance, Ca, Sr, Ba, Y, La-Nd, Sm, Gd-Dy, Er-Lu) (Yupko et al., 1974; Sakai et al., 1981b). Carbon dimers are particularly interesting due to the relationship between their lengths and the superconducting transition temperatures T_c_ (Kobayashi et al., 2019) of the compounds which feature these dimers. Since the C-C bond is quite short, the phonon frequency for the C-C stretching phonon modes is expected to be very high. Thus, for example, relatively high T_c_ values in La_2_C_3_ and Y_2_C_3_ [up to 13.4 K (Kim et al., 2007) and 18 K (Amano et al., 2004; Nakane et al., 2004), respectively] are ascribed to electron-phonon coupling between high-frequency phonons and C-C antibonding states at the Fermi level.

The lanthanide carbides family shows a large variety of possible phases with different stoichiometry at ambient pressure: RC_6_ (R = Eu), RC_2_ (R = Y, La-Lu), R_4_C_7_ (R = Y, Dy-Tm, Lu), R_2_C_3_ (La-Nd, Sm-Ho), R_3_C_4_ (Sc, Y, Tb-Lu), R_4_C_5_ (Y, Gd-Ho), R_4_C_3_ (R = Sc), RC_x_ (x ∼ 0.33-0.54, R = Sc, Y, Sm-Lu) (Babizhetskyy et al., 2017). Still, the number of known binary carbon compounds is significantly smaller than the number of known binary oxygen compounds (1,290 vs*.* 4,331) according to the ICSD database [Version 4.9.0 (build 20221006-1701)–Data Release 2022.2]. Considering that vast amount of the data corresponds to ambient conditions, the chemistry of carbides under high pressure has been poorly studied in principle. A recent discovery (Aslandukova et al., 2021) of the new γ-Y_4_C_5_ phase with non-linear [C_3_] groups synthesized above 40 GPa illustrates the importance of investigations of carbides at high pressures and motivates further studies of lanthanides carbides at non-ambient conditions. In this work, we report the high-pressure synthesis in laser-heated diamond anvil cells (DACs) of two novel carbides Dy_4_C_3_ and Dy_3_C_2_, and the formation of dysprosium sesquicarbide Dy_2_C_3_, already known at ambient conditions.

## 2 Experimental

The summary of all experiments is presented in Supplementary Table S1 (see Supplementary Information). In our experiments we used BX90-type diamond anvil cells with a large X-ray aperture (Kantor et al., 2012). As anvils we employed Boehler-Almax-type diamonds with culets diameter of 250 μm. Rhenium gaskets with an initial thickness of 200 μm were indented to ∼28 μm and a hole of ∼100 μm in diameter was drilled in the center of the indentation. Dysprosium flakes (99.9% purity, Merc Inc.) were loaded between one of the diamond anvils and a layer of dry sodium chloride (99.999% purity, ChemPUR) which played the role of a thermal insulator and a pressure transmitting medium; diamond anvils were used as a carbon source. Samples were compressed to the desired pressures and laser heated up to 2500 K. Laser heating of the samples was carried out using our *in house* double-sided YAG laser (1,064 nm wavelength) heating setup (Fedotenko et al., 2019). Thermal emission spectra from the heated area were collected using an IsoPlane SCT 320 spectrometer with a 1,024 × 2,560 PI-MAX 4 camera. Pressure was determined using the equation of states (EoS) of NaCl (Dorogokupets and Dewaele, 2007; Sakai et al., 2011).

The reaction products were analyzed by single-crystal X-ray diffraction (SCXRD) at several synchrotron beamlines: P02.2 of DESY, Hamburg, Germany (*λ* = 0.2894 Å, beam size ∼ 2 × 2 μm^2^) (Liermann et al., 2015); ID11 (*λ* = 0.2844 Å, beam size ∼ 0.75 × 0.75 μm^2^) and ID15B (*λ* = 0.4100 Å, beam size ∼ 1.5 × 2 μm^2^) of ESRF, Grenoble, France. During single-crystal data collection, the cell was rotated from −38° to +38° around the vertical ω axis with narrow 0.5° steps. XRD maps were created using the XDI software (Hrubiak et al., 2019) and helped to visualize the special distribution of various phases within the pressure chamber as well as to locate the areas where the step-scans should be performed. Powder XRD (PXRD) images were collected upon continuous rotation of the sample in a range of ±20° around the vertical ω axis at DESY, and ±1° around the vertical ω axis at ESRF. The CrysAlis^Pro^ software package (CrysAlisPRO) was used for the analysis of the single-crystal XRD data (peak hunting, indexing, data integration, frame scaling, and absorption correction). The DAFi program (Aslandukov et al., 2022) was used for the search of reflections’ groups belonging to individual single-crystal domains. Using the OLEX2 software package (Dolomanov et al., 2009), the structures were solved with the ShelXT structure solution program (Sheldrick, 2015b) using intrinsic phasing and refined with the ShelXL (Sheldrick, 2015a) refinement package using least-squares minimization. Crystal structure visualization was made with the VESTA software (Momma and Izumi, 2011).

The properties of the Dy_4_C_3_, Dy_2_C_3_, and Dy_3_C_2_ were determined through the first-principles calculations using the framework of density functional theory (DFT) as implemented in the VASP (Vienna *ab initio* simulation package) code (Kresse and Furthmüller, 1996). To expand the electronic wave function in plane waves we used the Projector-Augmented-Wave (PAW) method (Blöchl, 1994). The Generalized Gradient Approximation (GGA) functional was used for calculating the exchange-correlation energies, as proposed by Perdew–Burke–Ernzerhof (PBE) (Perdew et al., 1996). The PAW potentials with the following valence configurations of 5*s*5*p*6*s*5*d* for Dy and 2*s*2*p* for C were used to describe the interaction between the core and the valence electrons in frozen *f*-electrons approximation for Dy (Kresse and Furthmüller, 1996). Convergence tests with a threshold of 2 meV per atom in energy led to an energy cutoff for the plane wave expansion of 600 eV for all phases and a Monkhorst-Pack (Monkhorst and Pack, 1976) *k*-point grid of 4 × 4 × 4 for Dy_4_C_3_ and Dy_2_C_3_, and *k*-point grid of 5 × 5 × 9 for Dy_3_C_2_. Computations were performed for eight volumes that cover the pressure range of 0–100 GPa. Harmonic lattice dynamics calculations were performed with the PHONOPY software (Togo and Tanaka, 2015) using the finite displacement method for 2 × 2 × 2 (Dy_4_C_3_ and Dy_2_C_3_) and 2 × 2 × 3 (Dy_3_C_2_) supercells with respectively adjusted k-points. The tetrahedron method was used for Brillouin zone integrations, employing a mesh of 8 × 8 × 8 k-points for Dy_4_C_3_ and Dy_2_C_3_, and 10 × 10 × 18 k-points for Dy_3_C_2_ (Rath and Freeman, 1975; Friedrich, 2019). The integrated values of the crystal orbital bond index (ICOBI) (Müller et al., 2021) and Mulliken charges were calculated using LOBSTER v4.1.0 software (Maintz et al., 2016). The charge distribution in the ionic approximation based on a generalization of Pauling’s concept of bond strength (Pauling, 1929) was made using CHARDI 2015 (Nespolo and Guillot, 2016). In our calculations, temperature, configurational entropy, and the entropy contribution due to lattice vibrations were neglected.

## 3 Results

### 3.1 Structure of a novel dysprosium carbide Dy_4_C_3_

**FIGURE 1:**
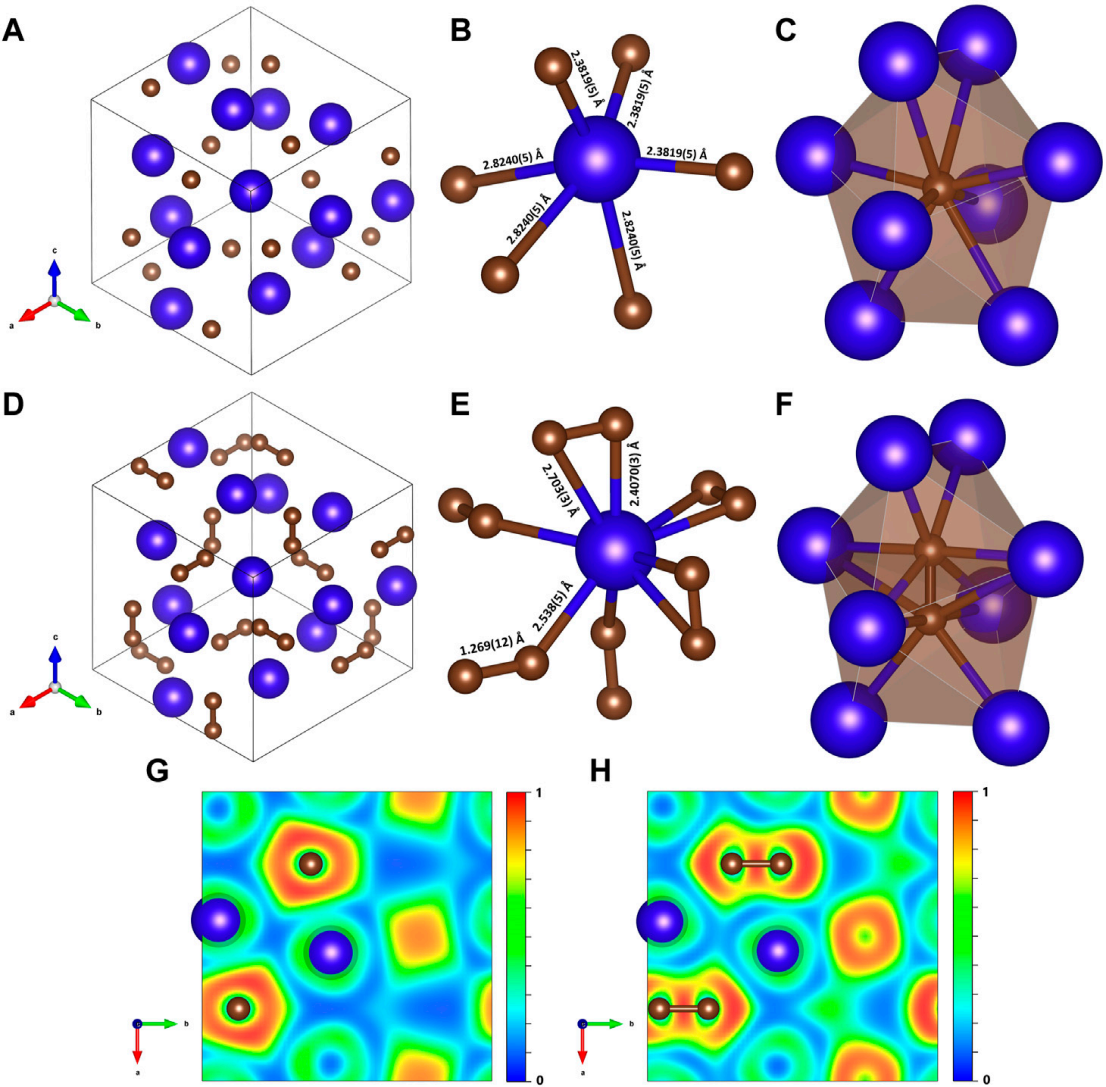
Crystal structures and corresponding 2D electron localization function (ELF) maps of Dy_4_C_3_ and Dy_2_C_3_ at 19 GPa. The blue and brown spheres represent dysprosium and carbon, respectively. Dy_4_C_3_: **(A)** Unit cell in projection along the [111] direction; **(B)** coordination polyhedron of Dy; **(C)** coordination environment of a carbon atom. Dy_2_C_3_: **(D)** Unit cell in projection along the [111] direction; **(E)** coordination polyhedron of Dy; **(F)** carbon dumbbell in a cage of eight Dy atoms. **(G)** and **(H)** are cross sections of the computed ELF shown in the (001) plane in Dy_4_C_3_ and Dy_2_C_3_, respectively.

The dysprosium carbide Dy_4_C_3_ was synthesized at 19, 55, and 58 GPa (Supplementary Table S1). This compound was hitherto unknown. It has the anti-Th_3_P_4_-type structure (space group *I*-43*d*) shown in Figures 1A–C, which has been described for scandium carbide, Sc_4_C_3_, but not observed in lanthanide carbides or Y carbides (Adachi et al., 1991; Babizhetskyy et al., 2017). At 19 GPa its unit cell parameter is equal to *a* = 7.4774 (8) Å.

In the structure of Dy_4_C_3_ (Figures 1A–C; Table 1), dysprosium and carbon atoms occupy the 16*c* and 12*a* Wyckoff sites, respectively (Supplementary Tables S2–S4). The coordination polyhedron of Dy cations is an irregular octahedron formed by the six nearest carbon atoms at distances of either 2.3819 (5) Å or 2.8240 (5) Å at 19 GPa (Figure 1B). Carbon atoms are surrounded by eight Dy atoms forming strongly distorted cubes (octaverticons) (Figure 1C).

**TABLE 1:** Selected experimental details of crystal structures of the carbides reported in this work.

Chemical formula	Dy_4_C_3_	Dy_2_C_3_	Dy_3_C_2_
Pressure (GPa)	19 (1)	19 (1)	55 (1)
Space group	*I-43d*	*I-43d*	*P4/mbm*
Space group number	#220	#220	#127
Structure type	anti-Th_3_P_4_	Pu_2_C_3_	U_3_Si_2_
a (Å)	7.4774 (8)	7.9208 (5)	5.9896 (13)
c (Å)			3.3880 (12)
V (Å^3^)	418.07 (13)	496.94 (9)	121.55 (7)
Z	4	8	2
R_int_	5.42%	2.85%	2.42%
R_1_	3.76%	1.50%	5.92%
No. of reflections	274	281	197
No. of parameters	7	11	11

### 3.2 Structure of dysprosium sesquicarbide Dy_2_C_3_

The cubic Dy_2_C_3_ sesquicarbide was synthesized in this work at 19 GPa (Supplementary Table S1). It has the Pu_2_C_3_-type structure (space group *I*-43*d*) with the unit cell parameter *a* = 7.9208 (5) Å at 19 GPa (Figures 1D–F; Table 1). The Dy_2_C_3_ sesquicarbide was earlier reported at ambient conditions with the lattice parameter equal to *a* = 8.198 (2) Å at 1 bar (Spedding et al., 1958). The dysprosium and carbon atoms occupy the 16*c* and 24*d* Wyckoff sites, respectively (Supplementary Table S5). The structure of Dy_2_C_3_ contains [C_2_] dumbbells with a length of ∼1.27 Å at 19 GPa.

The structures of Dy_2_C_3_ and Dy_4_C_3_ (described above) are closely related (see Figure 1): they have the same space group (*I*-43*d*), and the former can be easily derived from the latter, as the positions of the centers of [C_2_] dumbbells in Dy_2_C_3_ coincide with the positions of single carbon atoms in Dy_4_C_3_, whereas the coordinates of Dy atoms are the same in both structures. Thus, the coordination number of Dy atoms in Dy_2_C_3_ increases to nine (Figure 1E), whereas the coordination environment of [C_2_] dumbbells (Figure 1F) is similar to that of a single carbon atom in Dy_4_C_3_ (Figure 1C).

### 3.3 Structure of a novel dysprosium carbide Dy_3_C_2_

**FIGURE 2:**
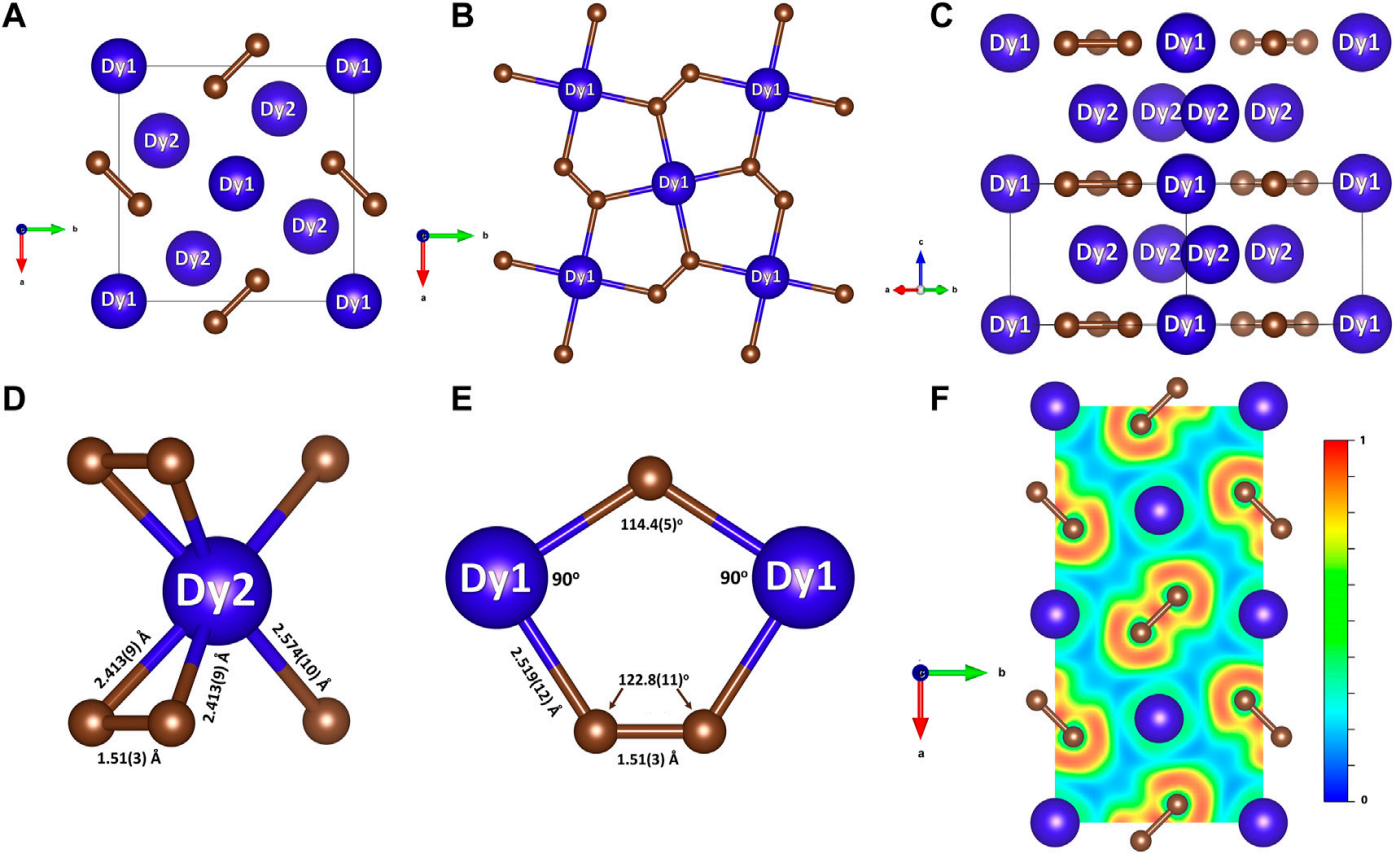
Crystal structure of Dy_3_C_2_ at 55 GPa and a cross section of the computed ELF. Blue and brown spheres represent dysprosium and carbon, respectively. **(A)** The structure viewed along the *c* direction; **(B)** the Dy1-C plane with the highlighted Cairo pentagonal tiling formed by Dy1 and C atoms; **(C)** the projection of the structure along the (110) direction highlighting the Dy1-C and Dy2 layers stacking in the *c* direction; **(D)** coordination of Dy2 atoms by carbon atoms; **(E)** interatomic distances in the Dy1-C plane; **(F)** the 2D ELF shown in the Dy1-C plane.

One more dysprosium carbide, Dy_3_C_2_, with a tetragonal unit cell (space group *P4/mbm*), was discovered at 55 GPa (Supplementary Table S1). At this pressure it has the following unit cell parameters: *a* = 5.9896 (13) Å, *c =* 3.3880 (12) Å (Figure 2; Table 1). Rare-earth metal carbides of such a stoichiometry have not been previously observed (Babizhetskyy et al., 2017), but the structure of the new Dy_3_C_2_ was found to be of the U_3_Si_2_-type, which is common for silicides (Zachariasen, 1948), borides (Riabov et al., 1999), and intermetallides (Chai and Corbett, 2011). Such structure was also theoretically predicted for a high-pressure calcium carbide Ca_3_C_2_ (Li et al., 2015).

In the structure of Dy_3_C_2_ (Figure 2; Table 1) carbon atoms occupying a single 4 *g* Wyckoff position and form [C_2_] dumbbells. Two dysprosium atoms are crystallographically distinct, occupying the Wyckoff positions 2*a* (Dy1) and 4*h* (Dy2). Figure 2A shows the structure of Dy_3_C_2_, as viewed along the *c* direction. Dy1 atoms lie in the same *ab* plane as the [C_2_] dumbbells, forming together the Cairo pentagonal tiling comprised of (Dy1)_2_C_3_ pentagons (Figure 2B). Such a structural motif is known in nickel diazenide NiN_2_, whose structure possesses atomic-thick layers comprised of Ni_2_N_3_ pentagons (Bykov et al., 2021), and in other compounds (Shao et al., 2018; Deng et al., 2020; Duan et al., 2022). Dy2 atoms are located in a parallel plane, separated from the described one by ½ *c* (Figure 2C). The Dy2-C inter-layer distances are of 2.413 (9) Å or 2.574 (10) Å (Figure 2D). As seen in Figure 2B, the Dy1 atoms are four-fold coordinated by C atoms with the Dy1-C distance equal to 2.519 (12) Å at 55 GPa. The length of the [C_2_] dumbbell is equal to 1.51 (3) Å (Figure 2E).

## 4 Discussion

### 4.1 Compressional behavior of Dy carbides

Dysprosium carbide Dy_4_C_3_ was synthesized at three different pressures (19, 55, and 58 GPa) that enabled us to analyse its structural response to compression. As expected, the shorter Dy-C contacts are less flexible than the longer ones: those being 2.3819 (5) Å and 2.8240 (5) Å at 19 GPa (Figure 1B) contract by ∼3.6% and ∼9.7%, respectively, upon compression to 58 GPa. The results of our DFT calculations agree well with the experimental data, suggesting ∼3.3% and ∼8.5%, correspondingly (Supplementary Tables S2–S4). Due to the anisotropy of compression, DyC_6_ polyhedra become less distorted with the distortion indices (D) equal to 0.085 and 0.053 at 19 and 58 GPa, respectively. A distortion index characterizes the average deviation of interatomic distances and angles from their mean values (Baur, 1974). *Ab initio* calculations reproduced well the experimental data with *D* = 0.084 at 19 GPa vs*. D* = 0.057 at 58 GPa.

**FIGURE 3:**
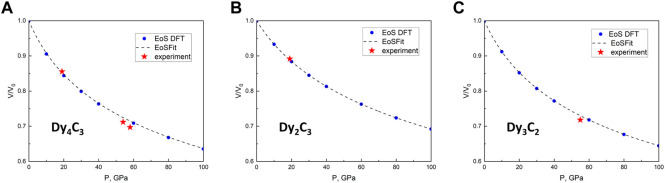
Pressure dependence of the relative volume for three Dy carbides. **(A)** Dy_4_C_3_, **(B)** Dy_2_C_3_ and **(C)** Dy_3_C_2_. The density functional theory (DFT)-calculated volumes for given pressures are shown by blue dots and dashed lines. Red stars indicate experimental data points.

In order to obtain the pressure dependence of the volume for the three dysprosium carbides and to determine the parameters of their equations of states (EOSes), we would need to measure volumes on decompression. However, as we performed our experiments in a solid pressure transmitting medium (NaCl), such data could not be reliable because of stresses. Due to that, we instead performed *ab initio* density functional theory (DFT) calculations in the pressure range up to 100 GPa. Their results are shown in Figure 3, and the parameters of the 3rd order Birch-Murnaghan (BM3) EOS, based on DFT calculations for Dy_2_C_3_, Dy_3_C_2_, and Dy_4_C_3_, are summarised in Table 2.

**TABLE 2:** Parameters of Birch-Murnaghan equation of state of studied dysprosium carbides obtained from *ab initio* calculations.

Compound	V_0_ (Å^3^)	K_0_ (GPa)	K´
Dy_4_C_3_	488.7 (7)	84.1 (11)	4.25 (3)
Dy_2_C_3_	557.28 (19)	125.8 (5)	4.169 (14)
Dy_3_C_2_	169.25 (18)	89.9 (9)	4.20 (3)

### 4.2 Charge analysis, bonding, and electronic properties

Dysprosium sesquicarbide Dy_2_C_3_ contains [C_2_] dumbbells with a length of ∼1.27 Å at 19 GPa, which is slightly shorter than the length of the double bond in ethylene and sesquicarbides Y_2_C_3_ and La_2_C_3_ at ambient pressure (Gready, 1984; Craig et al., 2006; Kim et al., 2007; Kobayashi et al., 2019). This suggests a formal charge of 4- for the [C_2_] dumbbell, so that the formula of Dy_2_C_3_ can be written as Dy^3+^_4_ [C_2_]^4–^_3_, and the compound can be called dysprosium (III) ethenide. The [C_2_] units in Dy_3_C_2_ have a length of ∼1.51 Å at 55 GPa, which is just a bit shorter than the C-C bond length in ethane (Gready, 1984). The compound may be described as Dy^2+^_3_ [C_2_]^6−^ and called dysprosium (II) ethanide. The Dy_4_C_3_ consists of single carbon atoms. With the formula Dy^3+^_4_C^4−^_3_ it is dysprosium (III) methanide.

In order to get a deeper insight into the crystal chemistry of the novel compounds, we performed a detailed charge and bond order analysis. Mulliken charge analysis (Müller et al., 2021) for the dysprosium atoms in carbides synthesized in this work yields the values of 1.62 in Dy_4_C_3_, 1.72 in Dy_2_C_3_, and 1.01 for Dy1 and 1.12 for Dy2 in Dy_3_C_2_ (Supplementary Table S7). The values for Dy_4_C_3_ and Dy_2_C_3_ are in agreement with Mulliken charges known for other dysprosium-containing compounds (Gupta et al., 2016; Ahuja et al., 2017). Our calculations of Mulliken charges for trivalent Dy carbides known at ambient conditions (DyC_2_ and Dy_4_C_5_ (Spedding et al., 1958; Adachi et al., 1976; Czekalla et al., 1997); are in a good agreement with those obtained for Dy_4_C_3_ and Dy_2_C_3_ (Supplementary Table S8). For Dy_3_C_2_, Mulliken charges of dysprosium are obviously lower, thus supporting our assessment of the cation in this compound as Dy^2+^ (see above). Notable is that at the same pressure of 55 GPa, the Dy-C distance in Dy_3_C_2_ is larger than in Dy_4_C_3_ (Supplementary Tables S3, S6), which also speaks in favor of a lower charge of dysprosium in the novel ethanide.

Assuming all Dy atoms to have integer charges, one can analyse carbon charges and the C-C chemical bonds in carbon dimers in different dysprosium carbides. The integrated crystal orbital bond indexes (ICOBI) obtained for DyC_2_ (Adachi et al., 1976), Dy_4_C_5_ (Czekalla et al., 1997), and Dy_2_C_3_ are close to 2 (Supplementary Table S8). The small deviations can be explained by shared-electron interactions due to the metallicity of the studied solids. This suggests a C=C double bond in these compounds, which is in a good agreement with its C-C distance (Supplementary Table S9). For Dy_3_C_2_ the ICOBI index for the C-C bond in the [C_2_] dimer differs significantly from those in other carbides (1.116) and suggests the bond order of 1, which is also consistent with the C-C bond length. Additionally, the assigned bond orders are well reflected in individual charges of C atoms and their anions, as obtained in both Mulliken and CHARDI approximations (Supplementary Table S8) (Nespolo and Guillot, 2016; Müller et al., 2021).

The character of the chemical bonding can be judged from calculated electron localization functions (ELF) (Savin et al., 1991). Relevant cross sections of ELFs at 19 GPa for Dy_4_C_3_ and Dy_3_C_2_ are shown in Figures 1G, H. They reveal ionic bonding between Dy and C in both compounds and strong covalent bonding in the [C_2_] dimers of Dy_2_C_3_. The 2D ELF for Dy_3_C_2_ at 55 GPa is shown in Figure 2F. It gives evidence of strong covalent bonding between carbon atoms in dimers and ionic bonds between Dy and C atoms.

**FIGURE 4:**
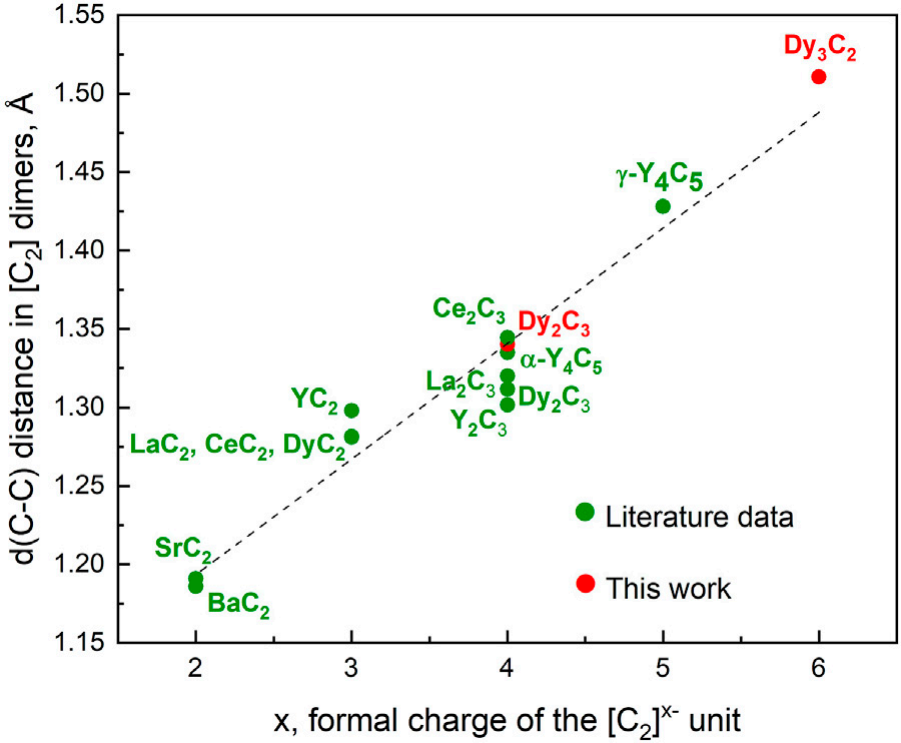
A correlation between the lengths of [C_2_] dimers, d (C-C), and their formal charges x in a number of binary metal-carbon compounds containing [C_2_]^x−^ species. All data corresponds to ambient pressure. Literature data are from experiments [(Atoji et al., 1958; Spedding et al., 1958; Atoji, 1967b; 1967a; Krupka et al., 1969; Adachi et al., 1976; Czekalla et al., 1997; Vohn et al., 2000; 1999; Yosida, 2002; Babizhetskyy et al., 2014; Aslandukova et al., 2021)], except for γ-Y_4_C_5_ (Aslandukova et al., 2021) and for Dy_2_C_3_ and Dy_3_C_2_ (this work, DFT-computed structures fully relaxed at 1 bar).

For 19 simple binary metal-nitrogen compounds containing [N_2_]^*x*–^species, a linear correlation was found between the length of the N–N dimers and their formal charges (Laniel et al., 2022). We used the literature data on 12 metal carbides studied at ambient conditions (Atoji et al., 1958; Spedding et al., 1958; Atoji, 1967b; 1967a; Krupka et al., 1969; Adachi et al., 1976; Czekalla et al., 1997; Vohn et al., 2000; 1999; Yosida, 2002; Babizhetskyy et al., 2014; Aslandukova et al., 2021) and our own results to analyse the relationship between the length of carbon dimers and their formal charges. For the novel Dy_3_C_2_, the C-C length at ambient pressure was obtained by DFT calculations, as well as for γ-Y_4_C_5_ in (Aslandukova et al., 2021); for Dy_2_C_3_ we included both the experimental value (Spedding et al., 1958) and the one DFT-calculated in this work, as they are a bit different. It appeared that the linear correlation holds also for carbides featuring [C_2_]^x−^ dimers at ambient conditions (Figure 4).

### 4.3 Vibrational properties and stability

**FIGURE 5:**
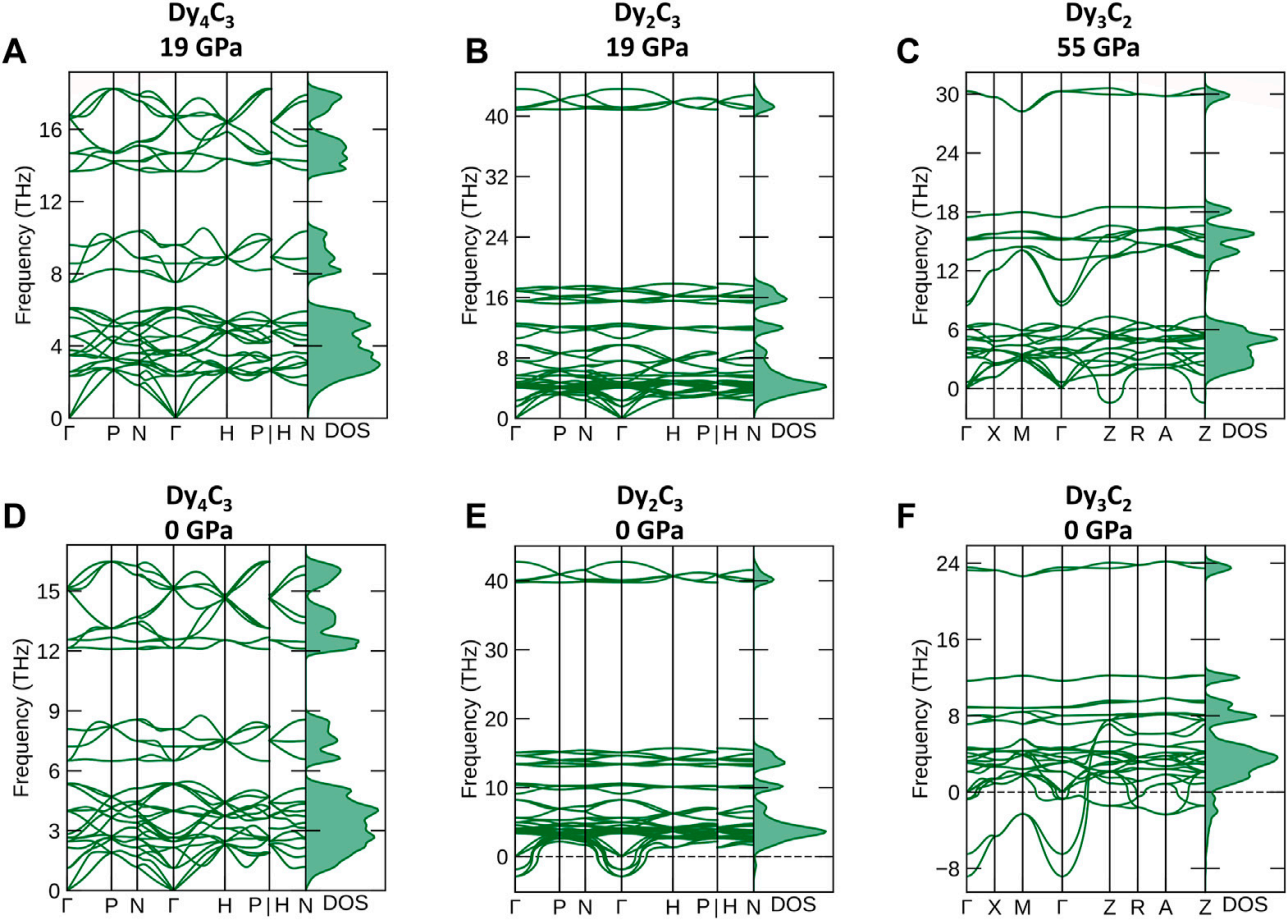
Phonon dispersion curves along high-symmetry directions in the Brillouin zone and phonon density of states. **(A)** Dy_4_C_3_, **(B)** Dy_2_C_3_ calculated at 19 GPa and **(C)** Dy_3_C_2_ at 55 GPa, **(D)** Dy_4_C_3_, **(E)** Dy_2_C_3_ and **(F)** Dy_3_C_2_ calculated at ambient pressure.

According to *ab initio* simulations of the phonon densities of state (pDOS) (Togo and Tanaka, 2015) in the harmonic approximation at 0 K, Dy_4_C_3_ and Dy_2_C_3_ compounds are dynamically stable at their synthesis pressure of 19 GPa (Figures 5A, B), whereas Dy_3_C_2_ is unstable both at its synthesis pressure of 55 GPa and at 1 bar (Figures 5C, F).

According to our calculations at 1 bar, Dy_2_C_3_ is not dynamically stable (Figure 5E), although it is known to exist at ambient conditions (Spedding et al., 1958). These inconsistency indicates some limitations of the theoretical analysis method we apply. Therefore, the predicted dynamical stability of Dy_4_C_3_ (Figure 5D) at ambient pressure should be considered with caution.

**FIGURE 6:**
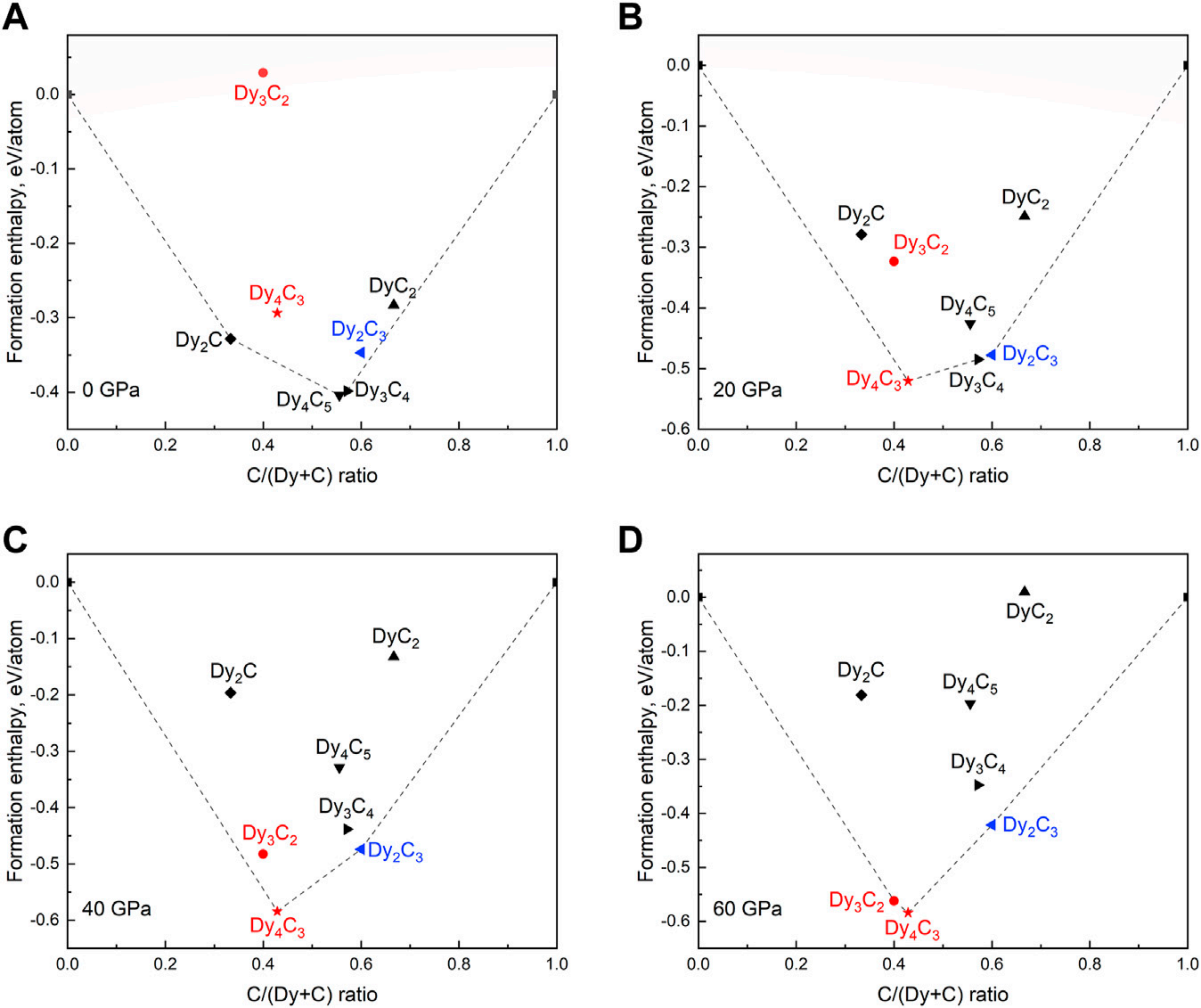
Calculated convex hull diagrams constructed for the Dy-C binary system for known dysprosium carbides. **(A)** 0 GPa, **(B)** 20 GPa, **(C)** 40 GPa, and **(D)** 60 GPa. Dashed lines indicate the convex hulls; carbides previously reported are marked by black symbols; carbides synthesized in this work are given in red and blue, indicating previously unknown and known, respectively.

To explore the thermodynamic stability of Dy_4_C_3_, Dy_2_C_3_, and Dy_3_C_2_ in comparison to other Dy carbides, convex hull diagrams were constructed considering known carbides [Dy_2_C (Atoji, 1981), Dy_4_C_5_ (Czekalla et al., 1997; Babizhetskyy et al., 2019; The Materials Project, 2020b), Dy_3_C_4_ (Hüfken and Jeitschko, 1998; The Materials Project, 2020a), DyC_2_ (Adachi et al., 1976)] at various pressures. Structure models for Dy_4_C_5_ (α-Y_4_C_5_ type) (The Materials Project, 2020b) and Dy_3_C_4_ (Sc_3_C_4_ type) (The Materials Project, 2020a) were acquired from Materials Project database, while those for Dy_2_C (Atoji, 1981) and DyC_2_ (Adachi et al., 1976)–from CIFs deposited in the ICSD database. The formation enthalpies were computed relative to the DFT total energies of the end-member elements Dy and C according to the equation: ΔH_f_ = (H_DyCx_—H_Dy_—x‧H_C_)/(1 + x). The results are shown in Figure 6. As seen, pressure has a very significant effect on the chemistry of the Dy-C system. Some phases (e.g., Dy_2_C and Dy_4_C_5_), which are stable at ambient pressure, become unstable already at 20 GPa. According to the convex hull diagram computed at 60 GPa, only those phases, which we observed in this work (Dy_4_C_3_, Dy_2_C_3_, and Dy_3_C_2_), are expected to be thermodynamically stable at such a high pressure.

## 5 Conclusion

The chemical reactions of dysprosium and carbon in diamond anvil cells at pressures of 19, 55, and 58 GPa and temperatures of ∼2500 K led to the synthesis of two novel dysprosium carbides, Dy_4_C_3_ at 19 GPa and Dy_3_C_2_ at 55 GPa, and one compound previously known at ambient condition, Dy_2_C_3_ at 19 GPa. The carbon atoms in the Dy_3_C_2_ and Dy_2_C_3_ form [C_2_] dumbbells, while there are single carbon atoms in Dy_4_C_3_. The crystal structure of Dy_4_C_3_ is of an anti-Th_3_P_4_ type. The structure of Dy_2_C_3_ can be derived from that of Dy_4_C_3_ if individual carbon atoms are replaced by dumbbells [C_2_]. Based on our new data, as well as literature data, we found a linear correlation between the formal charges of [C_2_]^x−^ groups and C–C interatomic distances. Theoretical calculations support our observations and also suggest that pressure drastically changes the chemistry of the Dy-C system.

## Data Availability

The datasets presented in this study can be found in online repositories. The names of the repository/repositories and accession number(s) can be found below: https://www.ccdc.cam.ac.uk/structures/-, 2248722, 2248721, 2248720, 2248679, and 2248647.
