# Supplementary material for: High-pressure synthesis of dysprosium carbides
Source: Front Chem. 2023 Jun 13;11:1210081. doi: 10.3389/fchem.2023.1210081 (PMC10296199; doi:10.3389/fchem.2023.1210081)
Supplement: Supplementary file 1 [file DataSheet1.PDF]

Supplementary Material

High-Pressure Synthesis of Dysprosium Carbides

Fariia Iasmin Akbar[1,2*], Alena Aslandukova[1], Andrey Aslandukov[1,2], Yuqing Yin[1,3], Florian Trybel[4], Saiana Khandarkhaeva[1], Timofey Fedotenko[5], Dominique Laniel[6], Maxim Bykov[7], Elena Bykova[2], Natalia Dubrovinskaia[1,4], Leonid Dubrovinsky[2]

[1]Material Physics and Technology at Extreme Conditions, Laboratory of Crystallography, University of Bayreuth, 95440 Bayreuth, Germany

[2]Bayerisches Geoinstitut, University of Bayreuth, 95440 Bayreuth, Germany

[3]State Key Laboratory of Crystal Materials, Shandong University, Jinan 250100, China

[4]Department of Physics, Chemistry and Biology (IFM), Linköping University 58183 Linköping (Sweden)

[5]Deutsches Elektronen-Synchrotron DESY, Notkestrasse 85, 22607 Hamburg, Germany

[6]Centre for Science at Extreme Conditions and School of Physics and Astronomy, University of Edinburgh, EH9 3FD Edinburgh, United Kingdom

[7]Institute of Inorganic Chemistry, University of Cologne, Greinstrasse 6, 50939 Cologne, Germany

*** Correspondence:** Fariia Iasmin Akbar: Fariia.Akbar@uni-bayreuth.de

Supplementary Tables

Supplementary Table S1. Summary of the high-pressure high-temperature experiments in diamond anvil cells.

DAC number	Starting materials	Culet size (μm)	Pressure (GPa, ±1)	Temperature (K, ±200)	Reaction products
1	Dy and NaCl	250	19	2500	Dy_2C_3, Dy_4C_3
2	Dy and NaCl	250	55	2500	Dy_3C_2, Dy_4C_3
3	Dy and NaCl	250	58	2500	Dy_4C_3

Supplementary Table S2. Experimentally determined crystallographic data for the Dy_4C_3 phase at 19 GPa in comparison to the corresponding DFT-relaxed structure. The pressure was fixed during the relaxation process, while the volume, cell shape, and atomic coordinates were allowed to vary freely. The full crystallographic dataset was deposited to the CCDC under the deposition number 2248720.

Chemical formula		Dy_4C_3 (experiment)	Dy_4C_3 (theory)
Pressure (GPa)		19(1)	19
Space group		*I*-43*d*	*I*-43*d*
Space group number		#220	#220
a (Å)		7.4774(8)	7.4638
V ($Å^3$)		418.07(13)	415.80
Z		4	4
CN of Dy		6	6
Dy-C distances in first coordination sphere (Å)		2.3819(5)-2.8240(5)	2.3827-2.8231
Atom / Wyck. site/ Fractional atomic coordinates (x; y; z)	Dy/16*c*	0.05589(5) 0.05589(5) 0.05589(5)	0.05636 0.05636 0.05636
	C/12*a*	0.375 0 0.25	0.375 0 0.25
R_{int}		5.42%	-
R_1		3.76%	-
No. of reflections		274	-
No. of parameters		7	-

Supplementary Table S3. Experimentally determined crystallographic data for the Dy_4C_3 phase at 55 GPa in comparison to the corresponding DFT-relaxed structure. The pressure was fixed during the relaxation process, while the volume, cell shape, and atomic coordinates were allowed to vary freely. The full crystallographic dataset was deposited to the CCDC under the deposition number 2248721.

<table>
<tr><td colspan="2">Chemical formula</td><td>Dy_4C_3 (experiment)</td><td>Dy_4C_3 (theory)</td></tr>
<tr><td colspan="2">Pressure (GPa)</td><td>55(1)</td><td>55</td></tr>
<tr><td colspan="2">Space group</td><td>I-43d</td><td>I-43d</td></tr>
<tr><td colspan="2">Space group number</td><td>#220</td><td>#220</td></tr>
<tr><td colspan="2">a (Å)</td><td>7.0335(13)</td><td>7.1099</td></tr>
<tr><td colspan="2">V ($Å^3$)</td><td>347.95(19)</td><td>359.41</td></tr>
<tr><td colspan="2">Z</td><td>4</td><td>4</td></tr>
<tr><td colspan="2">CN of Dy</td><td>6</td><td>6</td></tr>
<tr><td colspan="2">Dy-C distances in first coordination sphere (Å)</td><td>2.3046(9)-2.5760(9)</td><td>2.3179-2.6181</td></tr>
<tr><td rowspan="2">Atom / Wyck. site/ Fractional atomic coordinates (x; y; z)</td><td>Dy/16c</td><td>0.06548(10) 0.06548(10) 0.06548(10)</td><td>0.06379 0.06379 0.06379</td></tr>
<tr><td>C/12a</td><td>0.375 0 0.25</td><td>0.375 0 0.25</td></tr>
<tr><td colspan="2">R_{int}</td><td>2.74%</td><td>-</td></tr>
<tr><td colspan="2">R_1</td><td>3.84%</td><td>-</td></tr>
<tr><td colspan="2">No. of reflections</td><td>133</td><td>-</td></tr>
<tr><td colspan="2">No. of parameters</td><td>6</td><td>-</td></tr>
</table>

Supplementary Table S4. Experimentally determined crystallographic data for the Dy_4C_3 phase at 58 GPa in comparison to the corresponding DFT-relaxed structure. The pressure was fixed during the relaxation process, while the volume, cell shape, and atomic coordinates were allowed to vary freely. The full crystallographic dataset was deposited to the CCDC under the deposition number 2248722.

Chemical formula		Dy_4C_3 (experiment)	Dy_4C_3 (theory)
Pressure (GPa)		58(1)	58
Space group		*I*-43*d*	*I*-43*d*
Space group number		#220	#220
a (Å)		6.9846(6)	7.0434
V (Å^3)		340.74(9)	349.42
Z		4	4
CN of Dy		6	6
Dy-C distances in first coordination sphere (Å)		2.2954(8)-2.5499(8)	2.3047-2.5834
Atom / Wyck. site/ Fractional atomic coordinates (x; y; z)	Dy/16*c*	0.06648(10) 0.06648(10) 0.06648(10)	0.06503 0.06503 0.06503
	C/12*a*	0.375 0 0.25	0.375 0 0.25
R_{int}		3.17%	-
R_1		3.70%	-
No. of reflections		123	-
No. of parameters		6	-

Supplementary Table S5. Experimentally determined crystallographic data for the Dy_2C_3 phase at 19 GPa in comparison to the corresponding DFT-relaxed structure. The pressure was fixed during the relaxation process, while the volume, cell shape, and atomic coordinates were allowed to vary freely. The full crystallographic dataset was deposited to the CCDC under the deposition number 2248647.

Chemical formula		Dy_2C_3 (experiment)	Dy_2C_3 (theory)
Pressure (GPa)		19(1)	19
Space group		*I*-43*d*	*I*-43*d*
Space group number		#220	#220
a (Å)		7.9208(5)	7.9111
V (Å^3)		496.94(9)	495.12
Z		8	8
CN of Dy		9	9
Dy-C distances in first coordination sphere (Å)		2.4070(3)-2.703(3)	2.4137-2.7190
C–C distance in [C_2] dimers, Å		1.269(12)	1.333
Atom / Wyck. site/ Fractional atomic coordinates (x; y; z)	Dy/16*c*	0.04974(2) 0.04974(2) 0.04974(2)	0.05070 0.05070 0.05070
	C/24*d*	0.2949 0 0.25	0.29075 0 0.25
R_{int}		2.85%	
R_1		1.50%	
No. of reflections		281	
No. of parameters		11	

Supplementary Table S6. Experimentally determined crystallographic data for the Dy_3C_2 phase at 55 GPa in comparison to the corresponding DFT-relaxed structure. The pressure was fixed during the relaxation process, while the volume, cell shape, and atomic coordinates were allowed to vary freely. The full crystallographic dataset was deposited to the CCDC under the deposition number 2248679.

<table>
<tr><td colspan="2">Chemical formula</td><td>Dy_3C_2 (experiment)</td><td>Dy_3C_2 (theory)</td></tr>
<tr><td colspan="2">Pressure (GPa)</td><td>55(1)</td><td>55</td></tr>
<tr><td colspan="2">Space group</td><td>P4/mbm</td><td>P4/mbm</td></tr>
<tr><td colspan="2">Space group number</td><td>#127</td><td>#127</td></tr>
<tr><td colspan="2">a (Å)</td><td>5.9896(13)</td><td>5.9841</td></tr>
<tr><td colspan="2">c (Å)</td><td>3.3880(12)</td><td>3.4524</td></tr>
<tr><td colspan="2">V ($Å^3$)</td><td>121.55(7)</td><td>123.63</td></tr>
<tr><td colspan="2">Z</td><td>2</td><td>2</td></tr>
<tr><td colspan="2">CN of Dy1</td><td>4</td><td>4</td></tr>
<tr><td colspan="2">CN of Dy2</td><td>6</td><td>6</td></tr>
<tr><td colspan="2">Dy1-C distances in first coordination sphere (Å)</td><td>2.519(12)</td><td>2.5229</td></tr>
<tr><td colspan="2">Dy2-C distances in first coordination sphere (Å)</td><td>2.413(9)-2.574(10)</td><td>2.4215-2.6134</td></tr>
<tr><td colspan="2">C–C distance in [C_2] dimers, Å</td><td>1.51(3)</td><td>1.4828</td></tr>
<tr><td colspan="2">∠(Dy1-C-C), °</td><td>122.8(11)</td><td>123.01</td></tr>
<tr><td colspan="2">∠(Dy1-C-Dy1), °</td><td>114.4(5)</td><td>113.99</td></tr>
<tr><td colspan="2">∠(C-Dy1-C), °</td><td>90</td><td>90</td></tr>
<tr><td rowspan="3">Atom / Wyck. site/ Fractional atomic coordinates (x; y; z)</td><td>Dy1/2a</td><td>0 0 0</td><td>0 0 0</td></tr>
<tr><td>Dy2/4h</td><td>0.68222(14) 0.18222(14) 0.5</td><td>0.68053 0.18053 0.5</td></tr>
<tr><td>C/4g</td><td>0.089(2) 0.589(2) 0</td><td>0.08761 0.58761 0</td></tr>
<tr><td colspan="2">R_{int}</td><td>2.42%</td><td>-</td></tr>
<tr><td colspan="2">R_1</td><td>5.92%</td><td>-</td></tr>
</table>

No. of reflections	197	-
No. of parameters	11	-

Supplementary Table S7. Results of charge distribution analysis using Mulliken charge calculations based on the computed charge density and charge analysis in the ionic approximation for Dy_4C_3, Dy_2C_3 and Dy_3C_2 at synthesis conditions.

	Atom / Wyckoff site	Mulliken	CHARDI
Dy_4C_3 (55 GPa)	Dy/16*c*	1.62	2.94
	C/12*a*	-2.16	-3.92
Dy_2C_3 (19 GPa)	Dy/16*c*	1.72	2.97
	C/24*d*	-1.15	-1.98
Dy_3C_2 (55 GPa)	Dy1/2*a*	1.01	1.42
	Dy2/4*h*	1.12	2.46
	C/4*g*	-1.62	-3.17

Supplementary Table S8. Results of charge analysis for carbides containing [C_2] dumbbells which were either synthesised in this work (Dy_2C_3 and Dy_3C_2) or previously known (DyC_2 (Adachi et al., 1976) and Dy_4C_5 (Czekalla et al., 1997)) (Mulliken, CHARDI). The crystal orbital bond indexes (ICOBI) are given only for C-C bonds in [C_2] units. Structure model for Dy_4C_5 (α-Y_4C_5 type) (The Materials Project, 2020) was acquired from Materials Project database, while that for DyC_2 (Adachi et al., 1976) – from CIF deposited in the ICSD database.

	Atom / Wyckoff site	Fractional atomic coordinates (x; y; z)	Mulliken	Charge of [C_2]	ICOBI for C-C bond in [C_2]	CHARDI	Charge of [C_2]
Dy_2C_3	Dy/16*c*	0.04974(2) 0.04974(2) 0.04974(2)	1.72	-2.28	1.737	2.97	-3.96
	C/24*d*	0.2949 0 0.2500(7)	-1.15			-1.98	
Dy_3C_2	Dy1/2*a*	0 0 0	1.01	-3.24	1.116	1.42	-6.36
	Dy2/4*h*	0.68222(14) 0.18222(14) 0.5	1.12			2.47	
	C/4*g*	0.089(2) 0.589(2) 0	-1.62			-3.18	
DyC_2	Dy/2*a*	0 0 0	1.97	-1.96	2.115	2.96	-2.96
	C/4*e*	0 0 0.396	-0.98			-1.48	
Dy_4C_5	Dy1/4*h*	0.39239 0.30409 0.5	1.71	-2.22	1.893	3.36	-3.99
	Dy2/4*g*	0.24481 0.04280 0	1.54			2.67	
	C1/4*g*	0.09392 0.34397 0	-1.01			-2.08	
	C2/4*g*	0.13901 0.23568 0	-1.21			-1.91	
	C3/2*b*	0 0 0.5	-2.06			-4.08	

Supplementary Table S9. The C–C distances in [C_2] units in dysprosium carbides: Dy_2C_3 and Dy_3C_2 **-** synthesized in this work, DyC_2 (Adachi et al., 1976), and Dy_4C_5 (Czekalla et al., 1997). For calculations, the structure model for Dy_4C_5 (α-Y_4C_5 type) (Czekalla et al., 1997; The Materials Project, 2020) was acquired from Materials Project database, while that for DyC_2 (Adachi et al., 1976) – from CIF deposited in the ICSD database.

	Pressure (GPa)	C-C distance in [C_2] units, Å (experiment)	C-C distance in [C_2] units, Å (theory)
Dy_2C_3	19	1.269(12)	1.333
Dy_3C_2	55	1.51(3)	1.4828
DyC_2	0.0001	1.282	1.3029
Dy_4C_5	0.0001	1.3304	1.3330

Supplementary References

Adachi, G. Y., Shibata, Y., Ueno, K., and Shiokawa, J. (1976). Heats of the tetragonal-cubic transformation in rare earth dicarbides and mixed rare earth dicarbide solid solutions. *J. Inorg. Nucl. Chem.* 38, 1023–1026. doi: 10.1016/0022-1902(76)80021-8.

Czekalla, R., Hüfken, T., Jeitschko, W., Hoffmann, R. D., and Pöttgen, R. (1997). The Rare Earth Carbides R4C5 with R=Y, Gd, Tb, Dy, and Ho. *J. Solid State Chem.* 132, 294–299. doi: 10.1006/jssc.1997.7461.

The Materials Project (2020). Materials Data on Dy4C5 by Materials Project. *United States*. doi: https://doi.org/10.17188/1664795.
